# Nanowood: A Unique Natural Nanomaterial That Can Be Obtained Using Household Chemicals

**DOI:** 10.1021/acs.jchemed.4c00166

**Published:** 2024-10-10

**Authors:** Ievgen Nedrygailov, Darragh O’Brien, Scott Monaghan, Paul Hurley, Subhajit Biswas, Justin D. Holmes

**Affiliations:** † School of Chemistry, University College Cork, Cork T12 YN60, Ireland; ‡ AMBER Centre, Environmental Research Institute, University College Cork, Cork T23 XE10, Ireland; § Tyndall National Institute, University College Cork, Cork T12 R5CP, Ireland

**Keywords:** Upper-Division Undergraduate, Physical Chemistry, Hands-On Learning, Membranes, Nanofluidics, Electrochemistry

## Abstract

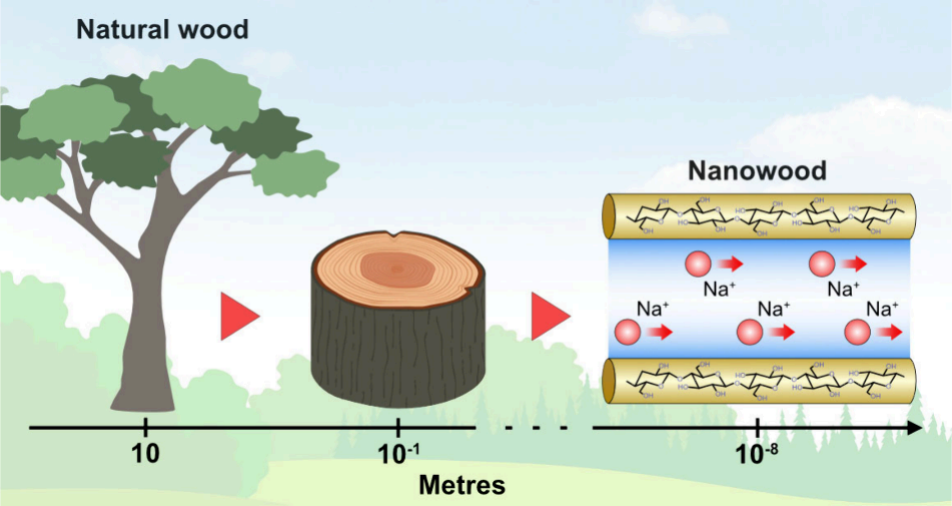

At the nanometer scale, electrolyte solutions behave differently compared to their bulk counterparts. This phenomenon forms the basis for the field of nanofluidics, which is dedicated to studying the transport of fluids within and around objects with dimensions of less than 100 nm. Despite the increasing importance of nanofluidics for a wide range of chemical and biochemical applications, the ability to study this field in undergraduate laboratories remains limited due to challenges associated with producing suitable nanoscale objects. This article outlines a straightforward procedure, using easily accessible materials and chemical reagents, to create nanofluidic membranes, called nanowood, containing channels with diameters less than 100 nm. We describe the fabrication process of nanofluidic channels in wood and demonstrate the presence of these nanochannels based on conductance measurements using electrochemical impedance spectroscopy.

## Introduction

When fluids are confined to a space whose dimensions do not exceed a hundred of nanometres (1 nm = 10^–9^ m), their properties undergo significant changes.^1,2^ For example, thermal conductivity, electrical conductivity, viscosity, chemical reactivity of electrolyte solutions placed in the space of nanopores, nanochannels or between close-packed colloidal nanoparticles with sizes between 1 and 100 nm may differ from those observed under normal conditions. Changes in the properties of confined fluids are a consequence of the interaction of solution ions with an electrical double layer (EDL) created by the surface charge. In nanopores and nanochannels, the dimensions of which are comparable to the thickness of the EDL, such interactions can be so intense that they can change the ionic composition of the fluid up to the almost complete exclusion of co-ions, which causes the observed changes in their properties. This phenomenon served as the basis for the creation of a new branch of science, called nanofluidics. Recently, nanofluidics has been widely used to separate ions and control their movement in various fields, including biology, chemistry, physics, and engineering.^3−5^

While there have been historical reports of unusual physical and chemical properties of liquids and gases confined within nanosized objects in various physical, chemical and biological experiments, the identification of nanofluidics as a separate branch of science took place only recently, about 30 years ago.^6^ This can be attributed to two key factors: i) the increasing demand for nanomaterials and nanodevices across a range of applications, including analytical separations, biomolecule analysis,^7,8^ lab-on-a-chip systems,^9^ sensors,^10^ electronic devices^11^ and energy harvesters^12−15^ and ii) significant advancements in nanotechnology, enabling the creation of nanofluidic devices with controlled geometry, size and properties. Typically, the creation of devices suitable for nanofluidic applications is carried out by highly qualified personnel using expensive equipment in clean-room conditions.^16−18^ Consequently, such devices are often not available for experiments in undergraduate laboratories. Nevertheless, certain natural materials, such as wood, inherently possess nanostructures. Therefore, the use of such materials in combination with simple chemical processing enables the production of nanofluidic devices without the need for specialized skills or equipment.^19−22^ Due to their accessibility, these devices can serve as an excellent teaching tool, giving students the opportunity to observe changes in the characteristic properties of electrolyte solutions during their transition from the bulk state to the nanoconfined state.

This study describes the fabrication process of nanofluidic devices called “nanowood membranes,” in which nanochannels with a diameter of less than 100 nm are created by delignifying natural wood using a household bleach solution. The existence of nanoconfined states of NaCl aqueous solutions of different concentrations in the nanowood membranes is demonstrated by measuring ionic conductance using electrochemical impedance spectroscopy.

## Pedagogical Goals

By completing this laboratory experiment, students are expected to gain a basic understanding of the phenomena that occur at liquid–solid interfaces on the nanometer scale, as well as the applications based on these phenomena. The specific pedagogical goals of this experiment include

- Getting to know wood as a unique natural nanomaterial.
- Gaining experience in delignification of natural wood to produce nanowood.
- Understanding the basic principles underlying nanofluidics.
- Understanding the fundamentals of ionic conductance of nanofluidic channels in electrolyte solutions of various concentrations.
- Understanding the fundamentals of electrochemical impedance spectroscopy.

Achievement of pedagogical goals is assessed by students’ responses to prelab questions (see Supporting Information), as well as by a written postlab report (see Results and Discussion).

## Experimental Section

### Nanowood Membranes

**Figure 1:**
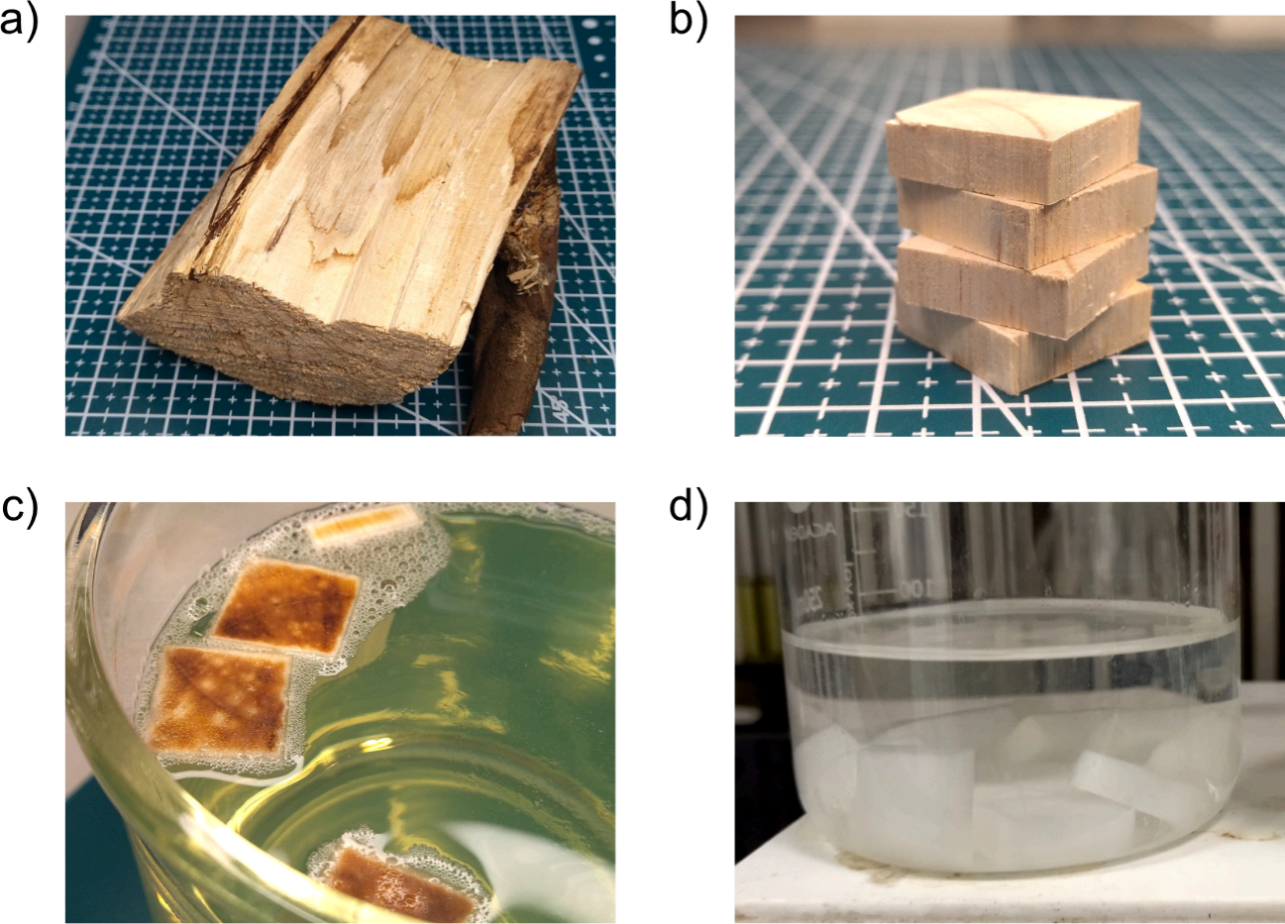
(a) A log of natural wood, used as a raw material for the manufacturing of nanowood membranes. (b) Wood blocks measuring 20 × 20 × 6 mm, after cutting the log with a saw. (c) Wood blocks on the first day after being placed in the household bleach solution. (d) Delignified wood blocks (nanowood membranes) in DI water.

A log of natural hardwood (Irish-grown *Eucalyptus Globulus*), similar to the one shown in Figure 1 (a), was used as a raw material for the production of nanowood membranes. Other types of hardwood can also be used as raw materials. Note that the composition of other types of hardwood (e.g., content of lignin and cellulose) may differ from the one used in this work. This may have an impact on the weight loss of the wood samples during the delignification process and conductance of the resulting membranes. However, such a change cannot affect the overall result of laboratory work. Before the laboratory activity, the log was cut into 20 × 20 × 6 mm blocks by qualified personnel using appropriate tools; see Figure 1 (b). These blocks were provided to students for delignification. The delignification process was carried out at room temperature for 7 days. In the first stage, the wood blocks with a total weight of 5 g were placed in a 2 L glass beaker with a household bleach solution containing 5% of sodium hypochlorite (NaClO); see Figure 1 (c). To mitigate any odor arising from the delignification process, the beaker was placed in a fume hood for the duration of the experiment. After 7 days, the wood blocks displayed a pristine white color. In the second stage, the delignified wood blocks (nanowood membranes) were removed from the bleach solution and washed in a beaker of warm deionized water (DI, 18.2 MOhm·cm) until the wash water reached a neutral pH; see Figure 1 (d). After washing, the nanowood membranes were placed in a container with DI water for storage.

### Measurements of Conductance

The conductance of nanowood membranes was measured in aqueous NaCl solutions of various concentrations (10^–6^–1 mol/L) using electrochemical impedance spectroscopy (EIS). We note that other methods can also be used to measure ionic conductance of nanowood membranes (see Supporting Information). To prepare a 1 mol/L NaCl solution, 58.44 g of pure NaCl from Sigma-Aldrich was placed in a 1 L volumetric flask. DI water was then added to the volumetric flask in two steps: 1) a small amount of water to completely dissolve the NaCl salt and 2) an additional volume of water to fill the volumetric flask to 1 L. Solutions of other concentrations were prepared from a 1 mol/L NaCl solution by diluting with DI water. To avoid contamination with ions of other types (e.g., calcium, potassium, chloride and sodium ions from tap water), all glassware was thoroughly rinsed with DI water before use.

**Figure 2:**
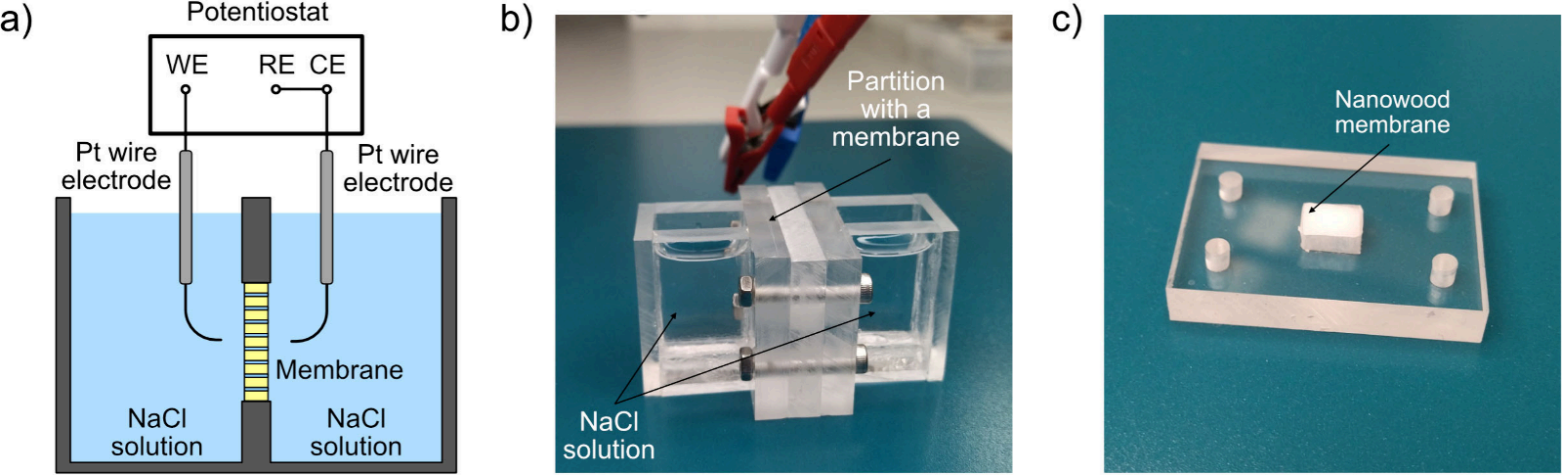
(a) Schematic representation and (b) photograph of a custom-built 2-electrode cell for measuring conductance of nanowood membranes using EIS. Here, WE is the working, RE is the reference and CE is the counter electrodes. (c) Sealed partition with a nanowood membrane before installation in the 2-electrode cell.

All measurements were carried out in a custom-built cell, as illustrated in Figure 2 (a,b). The cell was made of a 6 mm thick sheet of acrylic glass. A sealed partition was installed in the center of the cell, dividing it into two equal parts with a volume of approximately 5 mL each. A 6 × 8 mm aperture at the center of the partition was created to accommodate a nanowood membrane; see Figure 2 (c). The design ensured that the electric current between the electrodes (0.3 mm platinum wires) exclusively passes through the nanowood membrane. To reduce the series resistance associated with the NaCl solution, the distance from the membrane surface to each electrode was made small, on the order of 1 mm. Before using Pt electrodes to measure conductance of nanowood membranes, the electrodes were cleaned according to the following procedure. First, the electrodes were degreased in alcohol. Then, 50 cyclic voltammetry (CV) measurements were carried out in a glass beaker with 0.5 mol/L H_2_SO_4_ solution (BioLogic SP-50e potentiostat, scan rate: 20 mV/s, potential window: −0.2 to +1.2 V vs Ag/AgCl reference electrode). At the final stage, the electrodes were washed with DI water. The condition of the Pt electrodes after cleaning was checked by comparing the measured CV curves with literature data (see, for example, ref (23)).

EIS measurements were carried out in potentiostatic mode (PEIS) using the same BioLogic SP-50e potentiostat with an amplitude of 10 mV over a frequency range from 1 Hz to 200 kHz. All measurements were carried out at a potential of 0 V. Control of the potentiostat during measurements as well as analysis of measurement data was carried out using EC lab software from BioLogic. A vacuum was used to eliminate air bubbles and ensure complete saturation of the nanowood membranes with the electrolyte solution before EIS measurements. To do this, the cell shown in Figure 2 (b) with the installed membrane was immersed in a glass beaker filled with an electrolyte solution of the required concentration. Then the beaker with a cell inside was placed in a vacuum desiccator and the air was pumped out using a rotary vacuum pump. The procedure continued until air bubbles emerged from the nanowood membrane, which usually occurs within 10–15 min. At the end of the procedure, the cell was removed from the vacuum desiccator, cleaned of electrolyte residues on the outside, and connected to the potentiostat for measurements.

### Hazards and Safety Precautions

When working with a NaClO bleach solution, it is vital to follow the manufacturer’s safety instructions provided on the product packaging. In particular, the bleach solution should not be mixed with other chemicals to prevent potentially hazardous chemical reactions that could result in the release of toxic gases. All activities should be carried out in a fume hood or well-ventilated area. Personal protective equipment, including protective gloves, goggles and lab coats, should be worn at all times and contact of the bleach solution with bare skin, eyes, or mouth should be avoided. When planning experiments it is important to remember that the bleach solution may corrode certain metals and be harmful to aquatic life. Hence, the disposal of bleach solutions should adhere to established health and safety protocols.

EIS measurements should be carried out using appropriate equipment that is in good condition. All electrical connections must comply with safety regulations. In particular, touching the terminals or electrodes of the potentiostat with bare hands should be avoided to prevent electrostatic discharge. To minimize contamination with grease and organic matter, bare hand contact should be avoided with the cell, electrodes and membranes that have been cleaned and prepared for measurements.

## Results and Discussion

This laboratory activity was carried out with undergraduate chemistry students working individually as part of a final year research project and required two laboratory sessions held 1 week apart. However, it can also be done by students working in groups of two or three. The experiments can also be carried out with chemistry students of third and fourth years, as elective activities within the following modules: physical chemistry, analytical chemistry, nanomaterials and advanced research project. The laboratory work is carried out in two stages: 1) delignification of natural wood, 2) characterization of nanowood membranes.

### Prelab Assessment

Prelab reading covers the basics of 1) nanofluidics, 2) conductivity of nanochannels, and 3) electrochemical impedance spectroscopy (see Supporting Information). Prelab questions are used to assess students’ readiness to perform the laboratory work.

### Delignification of Natural Wood

**Figure 3:**
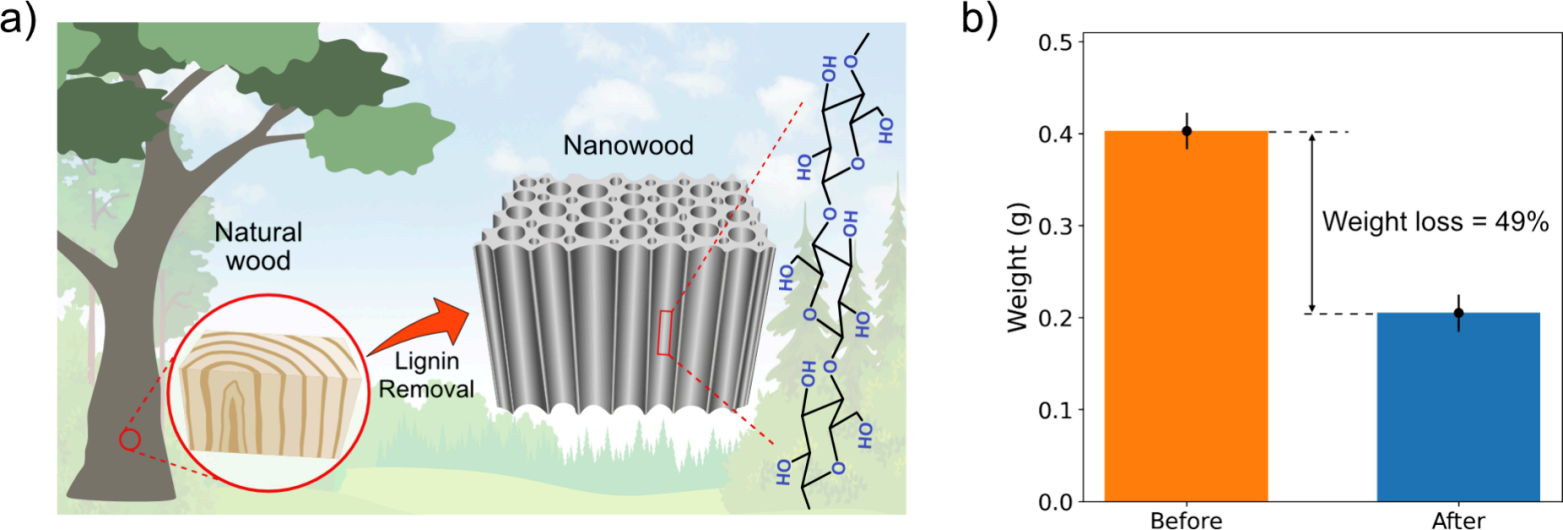
(a) Schematic illustration of the manufacturing of nanowood membranes. After lignin is removed, a nanowood is formed, which is a matrix of cellulose fibers forming a system of well-aligned nanofluidic channels. (b) Weight loss of eucalyptus wood samples after 7 days in a household bleach solution.

Natural wood has a unique hierarchical structure in which wood cells consist of cellulose fibers, hemicellulose and lignin. Using chemical processes known as delignification, lignin can be selectively removed from the cells, leaving a porous matrix with many straight, well-aligned nanochannels in the space between the cellulose fibers; see Figure 3 (a). In this way, natural wood can be converted into a nanofluidic membrane. Methods for treating wood cells may vary depending on the type of wood (e.g., hardwood or softwood), as described elsewhere.^19,24^ This experiment examined the simplest method for producing delignified wood using a household bleach solution.

To preliminary assess the condition of the wood blocks during the delignification process, their color was monitored. For the eucalyptus samples used in this experiment, a color change from pale cream to papery white occurred after approximately 7 days in the household bleach solution, compare Figure 1 (b) and Figure 1 (d). We note that the duration of delignification may vary depending on the type of wood and the household bleach solution used. Further analysis of the changes that occurred in the wood blocks as a result of delignification was carried out by comparing the weight of the blocks before and after exposure to the household bleach solution. To do this, three blocks were removed from the household bleach solution, washed in DI water, dried at a temperature of 70 °C until completely dry and weighed using a laboratory balances. The resulting weight was averaged and compared with the weight of the same blocks before the delignification process. As shown in Figure 3 (b), the blocks lost about 49% of their original weight, which is in good agreement with the lignin content in hardwood; see refs (19) and (24). The error bars in Figure 3 (b) indicate the standard deviation calculated using the method described in the Supporting Information.

### Measurements of Conductance

The weight loss of delignified wood blocks indicates the transition of wood to nanowood, which can act as nanofluidic membranes. The conductive properties of these membranes were assessed using EIS. To do this, the wood blocks extracted from the household bleach solution were washed in DI water, infiltrated in a vacuum with a NaCl solution of the required concentration, and then used for measurements in accordance with the procedure described in Experimental Section.

**Figure 4:**
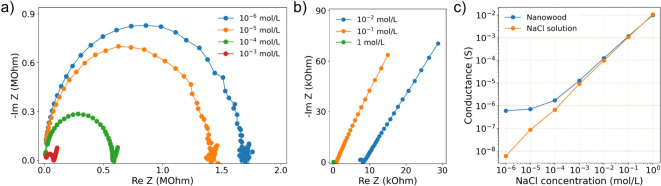
Typical Nyquist plots for nanowood membranes at (a) low and (b) high NaCl concentrations. (c) Conductance of a bulk electrolyte solution and a nanowood membrane at different NaCl concentrations.

Figure 4 (a,b) shows typical impedance spectra for nanowood membrane in NaCl solutions of various concentrations. For greater clarity, the results are divided into two graphs with different concentration ranges: 1) low concentration (*c* = 10^–6^–10^–3^ mol/L) and 2) high concentration (*c* = 10^–2^–1 mol/L). As seen, at low NaCl concentrations a Nyquist plot consists of a semicircle and a nonvertical straight line, inclined at an angle of about 45°. As the concentration of the solution increases, the diameter of the semicircle decreases. In addition, the whole curve shifts to the left, which corresponds to a decrease in the cell resistance. An even larger shift toward the low resistance region is observed in the case of high solution concentrations; see Figure 4 (b). In this case, the diameter of the semicircle becomes so small that, in fact, the entire impedance curve is just one nonvertical straight line.

EIS measurements using a cell without a membrane lead to Nyquist plots similar to those shown in Figure 4. However, the cell resistance values (*R*) deviate from the values obtained for a cell with the nanowood membrane. The difference between the measurement results using the cell with and without the nanowood membrane is shown in Figure 4 (c). To plot this graph, the cell resistance was determined for each solution. The cell conductance was then calculated based on these data as *G* = 1/*R* (see Supporting Information). As can be seen in Figure 4 (c), for a bulk NaCl electrolyte solution, the conductance of the cell is a linear (in log–log scale) function of the solution concentration over the entire measurement range. However, for the cell with the nanowood membrane, such a dependence is observed only at high concentrations of the electrolyte solution. At low concentrations the cell conductance becomes independent of the salt content in the solution and reaches a plateau. This behavior indicates a nanofluidic mode of membrane conductance,^1^ when the ion flow through the membrane is determined by the surface charge density in its nanochannels, and not by the bulk concentration of ions in the electrolyte (see Supporting Information).

### Postlab Assessment

This laboratory work was part of a BSc course in Chemistry and was conducted by individual fourth-year students. The effectiveness of achieving the pedagogical goals was evaluated through an analysis of written reports submitted by students at the end of the laboratory session. Each report included the following sections: title, abstract, introduction, materials and methods, results and discussion, conclusion, and references. The students’ knowledge was assessed based on their ability to explain key concepts in the introduction of the report (e.g., the concept of nanofluidics and its practical significance), description of laboratory procedures, and analysis of the measurement results. If necessary, an oral interview was conducted to clarify the student’s understanding based on their written report. After reviewing the outcomes of this laboratory work, including a survey of the student participants, the following conclusions were drawn:

1. All students demonstrated excellent results in all stages of the laboratory work, including prelab preparation, practical work in the laboratory, analysis of results, and report writing. The average overall result based on two years of data was at least 70%, which is considered excellent.
2. It was observed that the greatest challenges for students, both in preparing for the lab and in writing postlab reports, stemmed from understanding the theoretical foundations of electrochemical impedance spectroscopy. However, no difficulties were encountered in conducting electrochemical measurements or in analyzing the data. To address this issue, students were advised to consult additional literature on the fundamentals of electrochemical impedance spectroscopy (see refs 16 and 17 in the Student Handout).
3. All students unanimously highlighted the use of natural wood as a raw material for manufacturing nanofluidic membranes as the most interesting and inspiring aspect of this laboratory work.

## Conclusion

This study describes a laboratory experiment designed for undergraduate students aimed providing an understanding of fundamental concepts in nanofluidics. In particular, a simple process is described for producing nanofluidic membranes by delignifying natural wood using a household bleach solution. The conductive properties of the resulting membranes were investigated using electrochemical impedance spectroscopy measurements. In addition, a comparative analysis of the conductance of nanofluidic membranes and bulk NaCl electrolyte solutions at various NaCl concentrations was carried out. This analysis showed that the conductive properties of bulk electrolyte solutions coincide well with the classical theory: the conductance of solutions increases in proportion to their concentration. At the same time, the conductance of the nanofluidic membranes depends in a nontrivial way on the concentration of the electrolyte solution, which cannot be described in the same way as for bulk electrolyte solutions. At high concentrations, the conductance of nanofluidic membranes is described similarly to a bulk electrolyte. However, at low concentrations the conductance does not depend on the bulk properties of the electrolyte and is determined by the surface charge that arises in the nanochannels as a result of the formation of an electric double layer.

## Supporting Information Available

Instructor Notes: Additional Materials and Methods Information, Post-Lab Report Marking Rubric (PDF)
Instructor Notes: Additional Materials and Methods Information, Post-Lab Report Marking Rubric (DOCX)
Student Handout: Pre-Lab Reading, Pre-Lab Questions, Post-Lab Report Writing Guide, and Marking Scheme (PDF)
Student Handout: Pre-Lab Reading, Pre-Lab Questions, Post-Lab Report Writing Guide, and Marking Scheme (DOCX)
