# Supplementary material for: Nanowood: A Unique Natural Nanomaterial That Can Be Obtained Using Household Chemicals
Source: J Chem Educ. 2024 Oct 10;101(11):4931–6. doi: 10.1021/acs.jchemed.4c00166 (PMC11562577; doi:10.1021/acs.jchemed.4c00166)
Supplement: Supplementary file 1 — ed4c00166_si_001.pdf [file ed4c00166_si_001.pdf]

Supporting Information

Nanowood: A unique natural nanomaterial that can be obtained using household chemicals

Ievgen Nedrygailov[1,2*], Darragh O'Brien[1], Scott Monaghan[1,2,3], Paul Hurley[1,2,3], Subhajit Biswas[1,2] and Justin D. Holmes[1,2*]

[1]School of Chemistry, University College Cork, Cork, T12 YN60, Ireland

[2]AMBER Centre, Environmental Research Institute, University College Cork, T23 XE10, Ireland

[3]Tyndall National Institute, University College Cork, Cork, T12 R5CP, Ireland

Corresponding Authors
*E-mail: inedrygailov@ucc.ie (Ievgen Nedrygailov) and j.holmes@ucc.ie (Justin D. Holmes)

Materials and Methods

Ionic conductivity measurements

Electrochemical impedance spectroscopy is a very powerful and widely used method for studying processes in micro- and nanofluidic devices[1]. That is why this technique was chosen to measure the ionic conductivity of nanowood membranes in this work. However, in the absence of instruments allowing EIS measurements, the ionic conductivity of nanowood membranes can be determined using the following available methods, see Ref.[2–5]

Cell for measuring conductance

To perform this laboratory work, any standard electrochemical H-cell is suitable, in which the membrane is placed in the space between two chambers filled with an electrolyte solution of the same concentration and composition. The volume of the chambers may vary from a few millimeters to several tens of milliliters, depending on the laboratory supplies available and the volume of electrolyte solution available. In our case, a custom build cell was used. The cell was made from a sheet of acrylic glass and consisted of two rectangular chambers measuring 20x16x15 mm (approximate volume 5 mL), separated by a partition with a 6x8 mm window to accommodate the nanowood membrane.

Standard deviation

The standard deviation (σ) was calculated using the following equation:

$$\sigma = \sqrt{\frac{\sum_{i=1}^{n}(m_i - \overline{m})^2}{n-1}} \qquad \text{(S10)}$$

where m_i is weight of the ith sample, $\overline{m}$ is the mean sample weigh, and n is the number of samples.

Post-lab report marking rubric

The post-lab report is evaluated based on the following components:

- Abstract, aims and objectives (5 marks)
- Introduction – content and scope (25 marks)
- Structure and presentation (20 marks)
- Understanding and analysis (50 marks)
- **Total: 100 marks**

Each of the listed components is indicated as a percentage (see marking recommendations below). The overall score is obtained by scaling each individual component to the marks indicated above.

	**Outstanding ($\geq$ 80%)**	**Excellent (70 – 79%)**	**Very good (60 – 69%)**	**Good (50 – 59%)**	**Acceptable (40 – 49%)**	**Poor ($<$ 40%)**
	Exceptional across all categories	Excellent across all categories	Very good across all categories	Good across all categories	Adequate, though limited across all categories	Poor across all categories

**Abstract, Aims and Objectives (5 marks)**	Exceptional understanding of the initial aims of the lab work and the methods to be used to achieve goals	Excellent understanding of the initial aims of the lab work and the methods to be used to achieve goals	A very good understanding of the initial aims of the lab work and the methods to be used to achieve goals	A good understanding of the initial aims of the lab work and the methods to be used to achieve goals	Some understanding of the aims of the lab work and the methods to be used	Very poor understanding of aims of the lab work and of the methods to be used
**Introduction (25 marks)**	Exceptional level of content and scope showing an outstanding grasp of the field comparable to an expert	Excellent level of content and scope showing an excellent grasp of the field	Very good level of content and scope showing a very good understanding of the field	Good level of content and scope showing a good understanding of the field	Adequate level of content and scope showing some understanding of the field	No evidence of having consulted any primary sources and showing no understanding of the field
**Structure and presentation (20 marks)**	1.Exceptionally well-structured report<br>2.Content was exceptionally clear and accessible to the reader<br>3.Immaculately produced, virtually free from errors in grammar, spelling and punctuation<br>4.Figures, and tables exceptionally well-drafted<br>5.References are free from error	1.Very well-structured report<br>2.Content was very clear and readily accessible to the reader<br>3.Very well produced, with only very minor errors in grammar, spelling and punctuation<br>4.Figures and tables well drafted	1.Well-structured report<br>2.Clearly written apart from relatively minor aspects which do not seriously affect the understanding of the reader<br>3.Well produced, with only relatively minor errors in grammar, spelling and punctuation<br>4.Most figures and tables are well-drafted	1.Report lacked sufficient structure or clarity of expression in significant parts and would partially confuse or misguide a reader<br>2.Production standard is sufficient to allow the report to be understood	1.Poorly structured report<br>2.Writing style was unclear and presentation was such as to confuse and misguide the reader<br>3.There are many errors in punctuation, grammar and spelling which hinder the understanding of the reader<br>4.Figures and tables are poorly drafted and hard to read	1.The report was extremely disorganised and disjointed<br>2.The report was so badly produced as to severely limit the understanding of the reader
**Understanding and analysis (50 marks)**	1.Analysis of the findings is exceptionally clear and complete<br>2.The report demonstrates a full appreciation of the significance of the findings with respect to the specific objectives and in the wider context of the subject in general	1.Analysis of the findings is complete and very clear<br>2.The report demonstrates a very good appreciation of the significance of the findings with respect to the specific objectives as well as in the wider context of the subject in general.	1.A competent analysis of the findings is presented<br>2.The report demonstrates a good appreciation of the significance of the findings with respect to the specific objectives.	1.Some analysis of the findings and their context is presented.<br>2.The report demonstrates only a limited appreciation of the significance of the findings with respect to the project's objectives	1.The findings are presented with little or no analysis or discussion<br>2.The report shows no appreciation of the significance of the findings.	1.No analysis of the findings is presented<br>2.There is no appreciation of the significance of the findings and no evidence that the student had any understanding of the research undertaken.

References

(1) Schoch, R. B.; Han, J.; Renaud, P. Transport Phenomena in Nanofluidics. *Rev Mod Phys* **2008**, *80* (3), 839–883. https://doi.org/10.1103/REVMODPHYS.80.839/FIGURES/33/MEDIUM.

(2) Set, S.; Kita, M. Development of a Handmade Conductivity Measurement Apparatus and Application to Vegetables and Fruits. *J Chem Educ* **2014**, *91* (6), 892–897. https://doi.org/10.1021/ed400611q.

(3) Papadopoulos, N.; Limniou, M. A Computer-Controlled Bipolar Pulse Conductivity Apparatus. *J Chem Educ* **2001**, *78* (2), 245. https://doi.org/10.1021/ed078p245.

(4) Ghatee, M. H. A Simple Device for Conductivity Experiments. *J Chem Educ* **1993**, *70* (11), 944. https://doi.org/10.1021/ed070p944.

(5) Havrilla, J. W. An Inexpensive Device for Quantitative Conductivity Experiments. *J Chem Educ* **1991**, *68* (1), 80. https://doi.org/10.1021/ed068p80.
