# Supplementary material for: Nanowood: A Unique Natural Nanomaterial That Can Be Obtained Using Household Chemicals
Source: J Chem Educ. 2024 Oct 10;101(11):4931–6. doi: 10.1021/acs.jchemed.4c00166 (PMC11562577; doi:10.1021/acs.jchemed.4c00166)
Supplement: Supplementary file 4 — ed4c00166_si_004.docx [file ed4c00166_si_004.docx]

Supporting Information

Nanowood: A unique natural nanomaterial that can be obtained using household chemicals

Ievgen Nedrygailov^1,2*^, Darragh O'Brien^1^, Scott Monaghan^1,2,3^, Paul Hurley^1,2,3^, Subhajit Biswas^1,2^ and Justin D. Holmes^1,2*^

^1^School of Chemistry, University College Cork, Cork, T12 YN60, Ireland

^2^AMBER Centre, Environmental Research Institute, University College Cork, T23 XE10, Ireland

^3^Tyndall National Institute, University College Cork, Cork, T12 R5CP, Ireland

Corresponding Authors

*E-mail: [inedrygailov@ucc.ie](mailto:inedrygailov@ucc.ie) (Ievgen Nedrygailov) and [j.holmes@ucc.ie](mailto:j.holmes@ucc.ie) (Justin D. Holmes)

**Chapter 1: Before the Laboratory Work**

**Basics of nanofluidics**

Nanostructures consisting of channels with a characteristic size of less than 100 nm, i.e. nanochannels, have a very large surface area-to-volume ratio. Consequently, the interaction of a fluid, say, a liquid electrolyte, confined in the nanochannels with their walls can be very strong. This can dramatically change the ionic composition of the fluid up to the almost complete exclusion of co-ions and lead to significant changes in its properties, including changes in thermal and electrical conductivity, viscosity etc. Such influence of the nanochannels on the properties of fluids served as the basis for the creation of a new branch of science, called nanofluidics. The main goals of nanofluidics are 1) study and 2) control of the properties of fluids confined by nanochannels. Nanofluidics has a wide range of applications in various fields of biology, chemistry, physics and engineering including analytical separations, biomolecule analysis^1,2^, lab-on-a-chip systems^3^, sensors^4^, electronic devices^5^ and energy harvesters^6–9^.

To understand how the nanochannels affect the properties of fluids, it is necessary to consider the processes that occur when the fluid comes into contact with a solid surface. To be specific, let’s assume that our fluid is a solution of an aqueous electrolyte (e.g., NaCl solution) placed in a long channel. This means that the length of the channel ($L$) is significantly greater than its diameter ($H$), i.e. the condition $L\gg H$ is met. Also, we assume that the inner surface of the channel has an excess negative charge relative to the electrolyte solution. As will be described below, since the properties of the solid surface differ significantly from those of electrolytes, the formation of a solid-liquid interface (in our case, the boundary between the channel walls and the electrolyte solution) result in the appearance of a region whose conductivity differs significantly from that of the bulk electrolyte solution. Note that the formation of such a region with properties different from the bulk always occurs upon contact of a liquid and a solid. However, for large-scale objects encountered in everyday life and characterised by a low surface area-to-volume ratio, the contribution of the border region near the solid-liquid interface to the overall properties of the liquid is often insignificant. However, as the size of the object decreases, the influence of the surface becomes more noticeable. For nanoobjects, such as nanochannels, surface properties can dominate over the bulk properties.

Since the inner surface of the channel has an excess negative charge relative to the electrolyte solution, when they come into contact, an electric charge gradient occurs at the interface. To eliminate this gradient and bring the interface to electrical neutrality, cations in the solution migrate towards the surface, forming a positively charged layer, called the “Stern layer”. The resulting structure, consisting of a region of excess negative charge on the solid side and a compensating layer of excess positive charge on the electrolyte side (or vice versa), is called an electric double layer (EDL)^10,11^, see Figure S1 (a). The model of an EDL shown in Figure S1 (a) was originally proposed by physicist Hermann von Helmholtz back in 1853. Although this model provided a good basis for describing some important physical phenomena in electrolyte solutions, it fell short in describing the wide variety of properties exhibited by solid-liquid interfaces. In particular, Helmholtz’s model completely overlooked the disruptive influence of the thermal motion of ions, which can disperse the outer layer of charge^11^. Therefore, this model was improved in the subsequent works of Otto Stern, Louis Georges Gouy and David Leonard Chapman, who introduced the concept of a diffuse layer into the EDL model. In this improved version, known as the Stern-Gouy-Chapman model, the distribution of ion charges is adheres to Maxwell–Boltzmann statistics. Consequently, the electrical potential decreases exponentially with distance from the solid surface, as shown in Figure S1 (b). The electrical potential difference between the bulk of a liquid and a surface is called the surface potential. In this case, the characteristic thickness of the EDL, taking into account the diffusion layer, is determined by the Debye length ($\lambda_{D}$), which is defined by equation S1,

$\lambda_{D}=\sqrt{\frac{\varepsilon_{0}\varepsilon_{r}\mathrm{kT}}{2N_{A}\mathrm{Ie}^{2}}}=\frac{3.04\times{10}^{-10}}{z\sqrt{c}}$ (S1)

where $\varepsilon_{0}$ is the vacuum permittivity, $\varepsilon_{r}$ is the dielectric constant, $k$ is the Boltzmann’s constant, $T$ is the temperature, $N_{A}$ is the Avogadro constant, $I=\frac{1}{2}cz^{2}$ is the ionic strength of the electrolyte, $e$ is the elementary charge, $z$ is the valency of an ion and $c$ is the molar concentration. $\lambda_{D}$ exhibits an inverse relationship with the square root of the ion concentration. Therefore, in aqueous electrolyte solutions, such as NaCl solution, its value usually ranges from a few nanometres to several hundred nanometres, see Figure S1 (c).


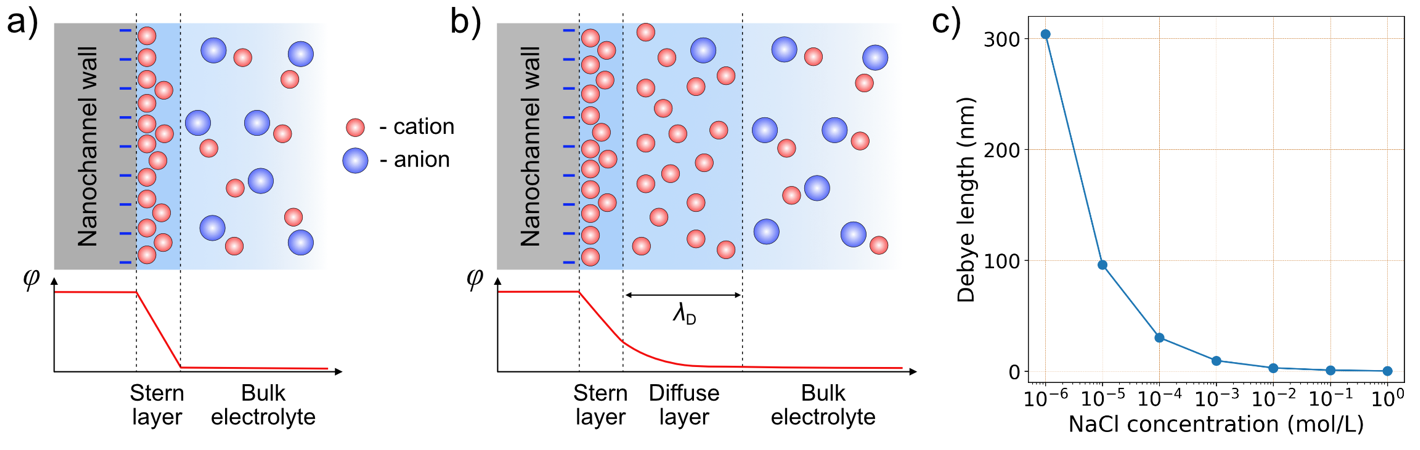


**Figure S1.** Schematic representation of the electric double-layer structure according to (a) Helmholtz model and (b) Stern-Gouy-Chapman model. The red curve shows the change in electrical potential ($\varphi$) with distance from the surface. (c) Dependence of the Debye length on NaCl concentration.

**Conductivity of nanochannels**

A simple yet powerful method for studying the processes occurring in a nanochannel filled with an electrolyte solution is to measure the ionic current that occurs when an electrical potential difference is applied between two electrodes located at opposite ends of the nanochannel under study, see Figure S2.


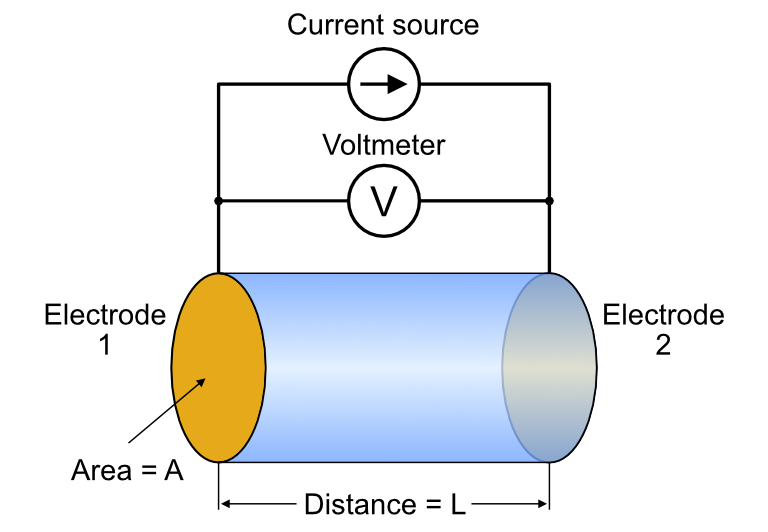


**Figure S2.** Scheme of a two-electrode setup for measuring the conductance of solutions.

When a potential difference $\Delta\varphi$ is applied to two electrodes at a distance $L$, the electric field causes the ions in the nanochannel to move. The resulting current ($J$) can be described by equation S2^10^,

$J=\frac{\Delta\varphi}{R}=G\Delta\varphi$ (S2)

where $G=1/R$ is the conductance of the nanochannel (the reciprocal of its resistance $R$). The conductance increases with the cross-sectional area ($A$) and decreases with the distance ($L$) between the electrodes. Therefore we can write

$G=\sigma\frac{A}{L}$ (S3)

where $\sigma$ is the nanochannel conductivity.

In general, the current through the nanochannel is the sum of two components: 1) the current in the bulk of the electrolyte solution and 2) the current along the surface of the nanochannel walls. Therefore, $J=J_{bulk}+J_{surface}$. In this case, the overall conductivity of the nanochannel, taking into account equations S2 and S3, is given by the sum of the bulk and surface conductivities

$\sigma=\sigma_{bulk}+\sigma_{surface}$ (S4)

The conductivity of the bulk electrolyte solution can be expressed as^10–12^,

$\sigma=\sigma_{bulk}={10}^{3}\left( \mu_{{Na}^{+}}+\mu_{{Cl}^{-}} \right)eN_{A}c$ (S5)

where $\mu_{{Na}^{+}}$ and $\mu_{{Cl}^{-}}$ are the mobilities of Na^+^ and Cl^-^ ions. By combining equations S3 and S5 the relation $G\propto c$ can be derived.

In turn, the surface conductivity of the nanochannel is determined by the surface charge density^10,12^, and ion transport occurs solely due to the movement of counterions. Consequently, the surface conductivity of the nanochannel can be expressed as

$\sigma_{surface}=\frac{2\sigma_{s}\mu_{{Na}^{+}}}{H}$ (S6)

where $H$ is the to the nanochannel diameter and $\sigma_{s}$ is the surface charge density^10,13–15^. By combining equations S3 and S6 the relation $G\propto\sigma_{s}$ can be derived.

Finally, the overall conductivity of the nanochannel, taking both components into account, is determined as

$\sigma=\sigma_{bulk}+\sigma_{surface}={10}^{3}\left( \mu_{{Na}^{+}}+\mu_{{Cl}^{-}} \right)eN_{A}c+\frac{2\sigma_{s}\mu_{{Na}^{+}}}{H}$ (S7)

For the purposes of this work, it is important to consider how the overall conductivity of the nanochannel, defined by equation S7, changes in electrolyte solutions of different concentrations.

As can be seen from Figure S1 (c), at high electrolyte concentrations (for our NaCl solution this corresponds to $c>{10}^{-3}$ mol/L) the Debye length is very small and amounts to only a few nanometres. Consequently, for nanochannels whose diameter is tens of nanometres (nanofluidic devices fall into this category), the condition ${2\lambda}_{D}\ll H$ will be met at such concentrations. In this case, the conductivity of the nanochannel does not differ from the conductivity of the bulk electrolyte solution. The ion current through the nanochannel includes both Na+ ions moving toward the negative electrode and Cl− ions being attracted to the positive electrode. The magnitude of the ion current at a constant nanochannel geometry and a constant potential difference depends only on the concentration of ions in the solution, see Figure S3 (a) and Figure S3 (b).

However, at low electrolyte concentrations ($c<{10}^{-3}$ mol/L), the EDL thickness becomes comparable to the diameter of the nanochannel or even exceeds it (i.e. ${2\lambda}_{D}\geq H$). In this case, the ion current through the nanochannel is determined by the movement of counterions only. Consequently, the conductivity of the nanochannel becomes more dependent on the surface charge density, see Figure S3 (a) and Figure S3 (c).

We note that if the condition ${2\lambda}_{D}\approx H$ is met, a transition state can be observed. In this case, the contributions from surface and bulk conductivity can be comparable, see Figure S3 (a). We also note that until now we have considered only the case of a single nanochannel. In practice, we also have to deal with nanofluidic membranes, which are an array of nanochannels. In the simplest case, one can determine the conductivity of such membranes as

$\sigma_{membrane}=\sigma N$ (S8)

where $N$ is the number of nanochannels in the membrane.


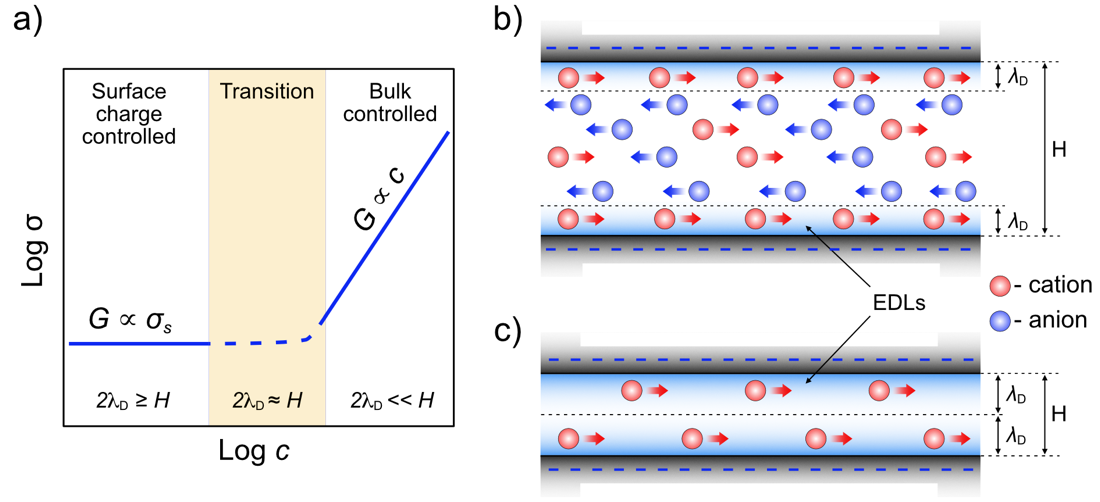


**Figure S3.** (a) Expected change in the overall conductivity of the nanochannel at different concentrations of electrolyte solution). Distribution of ions in the nanochannel in (b) bulk-controlled (2$\lambda_{D}\ll H$) and (c) surface-charge-controlled (${2\lambda}_{D}\geq H$) conditions.

**Basics of Electrochemical Impedance Spectroscopy**

As discussed in Sections 1 and 2, the conductive properties of a nanochannel change when its diameter is comparable to the thickness of the EDL. In the simplest case, the two-electrode setup shown in Figure S2 can be used to detect these changes. However, it should be taken into account that the mechanism of current flow in the measuring circuit and in the nanochannel is different. The current in the measuring circuit is caused by the movement of electrons along the metal wires, electrodes etc. In turn, the current in the nanochannel is caused by the movement of ions in the electrolyte solution. Therefore, it may be difficult to use direct current (DC) to measure nanochannel conductivity. Firstly, the ions are not able to leave the solution. Therefore, when DC potential difference is applied across the nanochannel, the ions will accumulate on the surface of the electrodes. This process creates additional barriers that affect the current through the nanochannel and lead to an error in measuring its conductivity. Secondly, electrolysis can occur on the surface of the electrodes. As a result, the concentration and composition of the electrolyte solution changes. This also leads to errors in measurements of nanochannel conductivity. To avoid these difficulties, it is convenient to use Electrochemical Impedance Spectroscopy (EIS). The essence of this method is to use a low-amplitude alternating signal (AC) to measure conductivity. In this section we provide only a brief overview of EIS. A more detailed description of the EIS measurement methodology and data analysis can be found elsewhere^16,17^.

Here we will limit ourselves to considering only two-electrode measurement scheme. However, in some cases, when carrying out high-precision measurements, 4 electrodes can also be used^16^. An input signal generated by an electrochemical workstation (potentiostat) obeys a sinusoidal law $x\left( t \right)=X_{0}\sin\left( \omega t \right)$, where $X_{0}$ is the amplitude and $\omega$ is the frequency, see Figure S4. Typically the EIS signal is an electrical potential. In this case, the measurement is carried out in potentiostatic mode (PEIS). However, in some cases (for example, when testing batteries), current can also be used as an input signal. In this case, EIS is carried out in galvanostatic mode (GEIS). As this signal passes through the electrochemical cell, both its phase and amplitude undergo changes, according to a relationship similar to Ohm's law for DC electrical circuits, specifically expressed as $J={\Delta\varphi}/Z$, where $J$ represents the current, $\Delta\varphi$ is the potential difference, and $Z$ is the total impedance of the electrochemical cell. Generally, the total impedance of the electrochemical cell can be written as

$Z=\sqrt{R^{2}+\left( X_{L}-X_{C} \right)^{2}}$ (S9)

where $R$ represents the ‘real’ component of the impedance (Re Z), signifying the value of ohmic losses of electrical energy when the EIS signal passes through the electrochemical cell. In turn, $X_{L}$ and $X_{C}$ are the ‘imaginary’ components of the impedance (Im Z), arising from inductive and capacitive energy losses.

To understand which components contribute to the total impedance of an electrochemical cell, the cell is tested using an input signal with constant amplitude but varying frequency over a certain range. The resulting spectrum of impedance values is then represented on a Nyquist plot, as exemplified in Figure S5. In this representation, the negative of the imaginary component of the total impedance is plotted against the real component of the total impedance. A detailed analysis of Nyquist plots is beyond the scope of this article and can be found elsewhere^16,17^. Of note, the intersection of the curve with the x-axis in the low frequency region signifies the total internal resistance of the cell. It encompasses the sum of the series resistance (*R_e_*), associated with the resistance of bulk electrolyte solution and the resistance *R_ct_*, which determines the diameter of the semicircle. The latter is associated with the so-called charge transfer resistance, or nanochannel resistance in the space between the electrodes, or the contact resistance of the electrode-electrolyte interface. In addition, after the semicircle a non-vertical line may be seen, which is associated with impedance due to ion diffusion in the bulk electrolyte or the channel. Therefore, by determining the point of intersection of the Nyquist plot with the x-axis in successive EIS measurements using the same electrochemical cell 1) first with only an electrolyte solution, then 2) with a nanochannel or membrane filled with the same electrolyte, it is possible to determine the resistance of the electrolyte and the membrane. Ultimately, using the resistance of the nanochannel or the membrane, its conductance can be determined as $G=1/R$.


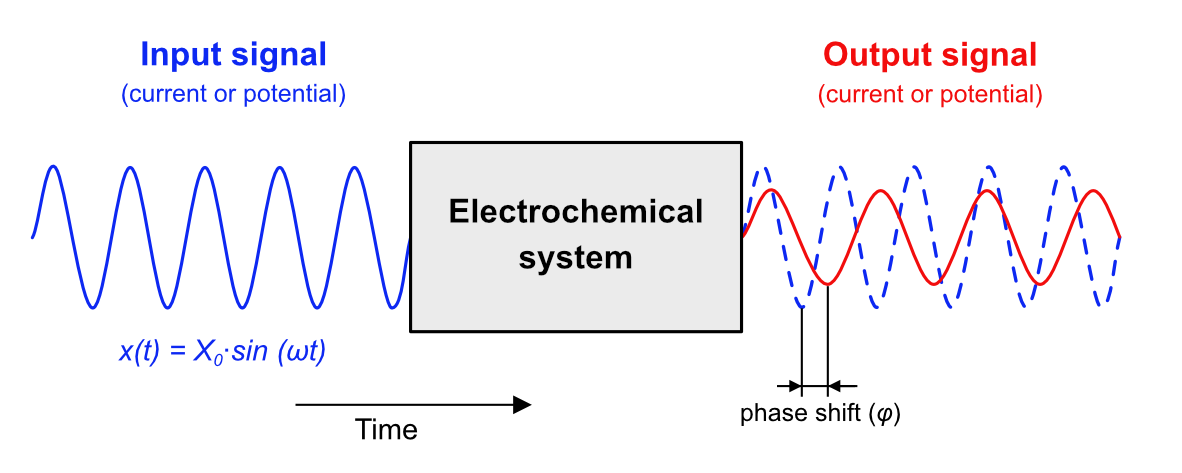


**Figure S4.** A simplified diagram showing the basic idea of electrochemical impedance spectroscopy. The input signal (current or potential, subject to a sinusoidal law $x\left( t \right)=X_{0}\sin\left( \omega t \right)$) is supplied to an electrochemical cell using a potentiostat. The output signal (current or potential) differs from the input signal in amplitude and phase. Analyzing the difference between the input and output signals allows one to assess the properties of the electrochemical system.


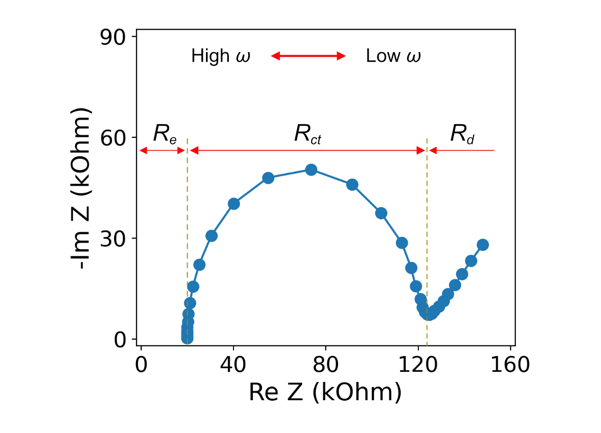


**Figure S5.** Schematic of a typical Nyquist plot. Here, R_s_ and R_ct_ denote the series resistance and charge transfer resistance of the electrochemical cell, respectively.

**Questions to answer before going to the lab:**

Before laboratory work, students should be familiar with the pre-lab readings and be able to answer the questions below. The criterion for admission to laboratory work is the ability to give correct answers to at least 5 out of 6 questions.

**Q1.** What is nanofluidics? What are its areas of application?

**Q2.** Define an electric double layer. Under what conditions is it formed and what models exist to describe the features of its structure?

**Q3.** Define the Debye length. How does the Debye length depend on the concentration of an electrolyte solution?

**Q4.** Define conductance and conductivity of a nanochannel. How does the conductivity of the nanochannel depend on the concentration of electrolyte solution?

**Q5.** Describe the basic idea of electrochemical impedance spectroscopy. Why does EIS use AC input signal instead of DC?

**Q6.** What is a Nyquist plot? How to use the Nyquist plot to determine the conductance of an electrochemical system?

**Chapter 2: After the Laboratory Work**

**Post-lab report**

Based on the results of the laboratory work, students are assigned to prepare a post-lab report. The report consist of the following sections: title, abstract, introduction, materials and methods, results and discussion, conclusion and references.

- **Title:** The title should be short and reflect the main purpose of the laboratory work.
- **Abstract:** The abstract should contain a brief (no more than 200 words) overview of the entire post-lab report, including purpose, methods and main findings.
- **Introduction:** The introduction should be written based on the pre-lab handouts and questions to them. It should present the background of nanofluidics, its research methods and applications as well as reasons for using wood in nanofluidics research. Also, the purpose and relevance of this laboratory work should be explained here.
- **Materials and methods:** This section details the materials used and the experimental procedures. It should also contain photographs or schematics of the experimental setup.
- **Results and discussion:** This section presents the main data obtained during the laboratory work. It may contain tables or graphs with measurement data and their analysis. During the discussion, these data should be analysed and conclusions drawn.
- **Conclusion:** The conclusion summarises the main findings and their implications. Also, it may suggest directions for future research or applications.

**Post-lab report marking scheme:**

**Total: 100 marks**

- Abstract, aims and objectives (5 marks)
- Introduction – content and scope (25 marks)
- Structure and presentation (20 marks)
- Understanding and analysis (50 marks)

**References**

(1) Yamamoto, K.; Ota, N.; Tanaka, Y. Nanofluidic Devices and Applications for Biological Analyses. *Anal Chem* **2021**, *93* (1), 332–349. https://doi.org/10.1021/ACS.ANALCHEM.0C03868/ASSET/IMAGES/LARGE/AC0C03868_0008.JPEG.

(2) Rathnayaka, C.; Amarasekara, C. A.; Akabirov, K.; Murphy, M. C.; Park, S.; Witek, M. A.; Soper, S. A. Nanofluidic Devices for the Separation of Biomolecules. *J Chromatogr A* **2022**, *1683*, 463539. https://doi.org/10.1016/J.CHROMA.2022.463539.

(3) Verma, N.; Pandya, A. Challenges and Opportunities in Micro/Nanofluidic and Lab-on-a-Chip. *Prog Mol Biol Transl Sci* **2022**, *186* (1), 289–302. https://doi.org/10.1016/BS.PMBTS.2021.07.016.

(4) Hu, Y. L.; Cui, H. S.; Yu, C. M.; Wu, Z. Q. Nanofluidic Electrochemical Sensors for Clinical Biomarkers Detection. *Microchemical Journal* **2023**, *193*, 109058. https://doi.org/10.1016/J.MICROC.2023.109058.

(5) Karnik, R.; Fan, R.; Yue, M.; Li, D.; Yang, P.; Majumdar, A. Electrostatic Control of Ions and Molecules in Nanofluidic Transistors. *Nano Lett* **2005**, *5* (5), 943–948. https://doi.org/10.1021/NL050493B/SUPPL_FILE/NL050493BSI20050314_114818.PDF.

(6) Laucirica, G.; Toimil-Molares, M. E.; Trautmann, C.; Marmisollé, W.; Azzaroni, O. Nanofluidic Osmotic Power Generators – Advanced Nanoporous Membranes and Nanochannels for Blue Energy Harvesting. *Chem Sci* **2021**, *12* (39), 12874–12910. https://doi.org/10.1039/D1SC03581A.

(7) Hamzat, A. K.; Omisanya, M. I.; Sahin, A. Z.; Ropo Oyetunji, O.; Abolade Olaitan, N. Application of Nanofluid in Solar Energy Harvesting Devices: A Comprehensive Review. *Energy Convers Manag* **2022**, *266*, 115790. https://doi.org/10.1016/J.ENCONMAN.2022.115790.

(8) Guo, W.; Cao, L.; Xia, J.; Nie, F. Q.; Wen, M.; Xue, J.; Song, Y.; Zhu, D.; Wang, Y.; Jiang, L. Energy Harvesting with Single-Ion-Selective Nanopores: A Concentration-Gradient-Driven Nanofluidic Power Source. *Adv Funct Mater* **2010**, *20* (8), 1339–1344. https://doi.org/10.1002/ADFM.200902312.

(9) Xiao, K.; Jiang, L.; Antonietti, M. Ion Transport in Nanofluidic Devices for Energy Harvesting. *Joule* **2019**, *3* (10), 2364–2380. https://doi.org/10.1016/J.JOULE.2019.09.005.

(10) Morgan, H.; Green, N. G. *AC Electrokinetics : Colloids and Nanoparticles*; Research Studies Press, 2003.

(11) Atkins, P. J.; Paula, J. D.; Keeler, J. *Atkins Physical Chemistry*; Oxford Univ. Press: Oxford, 2017.

(12) Martins, D. C.; Chu, V.; Conde, J. P. The Effect of the Surface Functionalization and the Electrolyte Concentration on the Electrical Conductance of Silica Nanochannels. *Biomicrofluidics* **2013**, *7* (3). https://doi.org/10.1063/1.4811277.

(13) Abgrall, P.; Nguyen, N. T. Nanofluidic Devices and Their Applications. *Anal Chem* **2008**, *80* (7), 2326–2341. https://doi.org/10.1021/AC702296U/ASSET/IMAGES/LARGE/AC702296UF00008.JPEG.

(14) Schoch, R. B.; Renaud, P. Ion Transport through Nanoslits Dominated by the Effective Surface Charge. *Appl Phys Lett* **2005**, *86* (25), 1–3. https://doi.org/10.1063/1.1954899/330184.

(15) Schoch, R. B.; Han, J.; Renaud, P. Transport Phenomena in Nanofluidics. *Rev Mod Phys* **2008**, *80* (3), 839–883. https://doi.org/10.1103/REVMODPHYS.80.839/FIGURES/33/MEDIUM.

(16) Lazanas, A. C.; Prodromidis, M. I. Electrochemical Impedance Spectroscopy─A Tutorial. *ACS Measurement Science Au* **2022**, *2023*, 162–193. https://doi.org/10.1021/ACSMEASURESCIAU.2C00070/ASSET/IMAGES/LARGE/TG2C00070_0032.JPEG.

(17) Kemp, N. T. A Tutorial on Electrochemical Impedance Spectroscopy and Nanogap Electrodes for Biosensing Applications. *IEEE Sens J* **2021**, *21* (20), 22232–22245. https://doi.org/10.1109/JSEN.2021.3084284.
